# The Survival Outcomes of the Metastatic Nonclear Cell Renal Cell Carcinoma in the Immunotherapy Era: Princess Margaret Cancer Centre Experience

**DOI:** 10.15586/jkcvhl.v11i1.307

**Published:** 2024-03-02

**Authors:** Esmail Al-Ezzi, Abhenil Mittal, Zachary W. Veitch, Amer Zahralliyali, Nely Mercy Diaz Mejia, Osama Abdeljalil, Husam Alqaisi, Vikaash Kumar, Aaron R. Hansen, Nazanin Fallah-Rad, Srikala S. Sridhar

**Affiliations:** 1 Division of Medical Oncology and Hematology, Princess Margaret Cancer Centre, University Health Network, Toronto, ON, Canada;; 2 Division of Medical Oncology and Hematology, Royal Victoria Hospital, Barrie, ON, Canada;; 3 Division of Cancer Services, Princess Alexandra Hospital, Metro South Health, Brisbane, QLD 4113, Australia

**Keywords:** immunotherapy, kidney cancer, nonclear cell, survival outcomes, targeted therapy

## Abstract

Immunotherapy (IO) with or without targeted therapy (TT) is the standard treatment for patients with metastatic clear cell renal cell carcinoma (RCC). The evidence supporting their use in metastatic nonclear cell renal cell carcinoma (nccRCC) subtypes is based on small prospective trials and retrospective analyses. Here, we report survival outcomes for patients with metastatic nccRCC treated with IO and/or TT at the Princess Margaret Cancer Centre, Toronto, ON, Canada. Demographics, disease characteristics, and survival outcomes were collected retrospectively. Overall (OS), progression-free survival (PFS), and objective response rates (ORR) were calculated. We identified 69 patients with metastatic nccRCC treated with IO and/or TT as the first-line treatment, and 36 (52.1%) patients as the second-line treatment. Median OS of the first line IO recipients (n = 12) and non-IO recipients (n = 57) was not reached (NR) and 17.2 months (95% confidence interval (95% CI): 7.3–27.0; P = 0.23), respectively. Median PFS of first-line IO recipients and non-IO recipients was NR and 4.7 months (95% CI: 3.7–5.6; P = 0.019), respectively. The ORR of IO recipients versus non-IO recipients was 50% versus 12.3% (P = 0.007). Median OS of the second-line IO recipients (n = 8) and non-IO recipients (n = 28) was NR and 6.3 months (95% CI: 3.2–9.3; P = 0.003), respectively. Median PFS of second-line IO recipients and non-IO recipients was 4.8 months (95% CI: 2.7–6.8) and 2.8 months (95% CI: 1.8–3.7; P = 0.014), respectively. ORR of IO recipients and non-IO recipients was 37.5% and 3.5%, respectively; P = 0.028. While the number of patients included in our retrospective review was small, our analysis suggested that patients with nccRCC have improved survival outcomes with IO treatment. Validation of prospective dataset is required before widespread clinical utilization.

## Introduction

Kidney cancer is the 14th most common cancer worldwide with an estimated 430,000 new cases and 179,368 deaths in 2020 (1). It was estimated that 8100 persons in Canada would be diagnosed with kidney cancer, and 1950 in Canada would die from it in 2022 (2). Renal cell carcinoma (RCC) is the most common kidney tumor in adults. The predominant histological subtype of RCC is clear cell type (ccRCC), accounting for approximately 70–90% of cases. This is followed by nonclear cell subtypes (nccRCC), including papillary (10–15%) and chromophobe (3–5%) subtypes (3). XP11.2 translocation RCC is a relatively uncommon and aggressive variant of nccRCC, comprising 0.72–1.6% of adult RCC patients. It is distinguished by chromosomal rearrangements involving the *TFE3* gene (4). Unclassified RCC refers to renal tumors that do not conform to any established subtypes. These tumors account for 2–6% of all RCC patients (5). NccRCC subtypes exhibit notable differences in terms of their biological characteristics, clinical behavior, and prognostic outcomes, compared to ccRCC (6). Many studies have documented superior survival rates and a more favorable prognosis in patients with localized nccRCC, compared to ccRCC (7). On the contrary, inferior survival outcomes have been reported in patients who have metastatic papillary type II and XP11.2 translocation (8).

The majority of prior randomized clinical trials have focused on investigating the efficacy of targeted therapy (TT) in treating nccRCC. This includes the use of vascular endothelial growth factor receptor (VEGFR) tyrosine kinase inhibitors, mammalian target of rapamycin (mTOR) inhibitors, and mesenchymal-epithelial transition (MET) kinase inhibitors. ASPEN was a randomized multicenter clinical trial that enrolled 108 patients with advanced nccRCC to either sunitinib or everolimus group. Progression-free survival (PFS) was significantly increased with sunitinib (8.3 months [95% confidence interval, 95% CI: 5.8–11.4] vs. 5.6 months [95% CI: 5.5–6.0]; P = 0•16) (9). Another phase II randomized clinical trial compared sunitinib with other tyrosine kinase inhibitors, such as cabozantinib, crizotinib, and savolitinib for the treatment of advanced papillary renal carcinoma. Cabozantinib showed significant PFS improvement, compared to sunitinib (median 9.0 months, 95% CI: 6–12, vs. 5.6 months, 95% CI: 3–7; P = 0.019). Objective response rate (ORR) for cabozantinib was 23% versus 4% for sunitinib (P = 0.010). Savolitinib and crizotinib failed to show improvement in PFS, compared to sunitinib (10).

The management of ccRCC with combination immunotherapy (IO) and IO–TT combination approaches is based on a number of randomized clinical trials comparing these treatments with sunitinib (11–15). However, only a small number of patients with nccRCC were included in these studies, and definite conclusions about the efficacy of these regimens in these subgroups were difficult to ascertain. Currently, the role of IO in the treatment of advanced nccRCC patients is based on smaller prospective trials and retrospective analyses with different survival outcomes, posing a challenge to oncologists to treat and counsel such patients (16). Therefore, real-world data on the efficacy of IO-based treatments for these rare subtypes is essential for treatment planning and counseling. A phase III randomized trial comparing the IO combination (nivolumab + ipilimumab) versus sunitinib in nccRCC is ongoing (NCT03075423).

Herein, we report a real-world experience of the survival outcomes of patients with nccRCC treated at the Princess Margaret Cancer Centre, Toronto, ON, Canada with IO and/or TT in the first- and second-line treatment settings.

## Patients and Methods

### Patients and data collection

Patients with metastatic nccRCC treated with IO and/or TT at our cancer center from July 2002 to July 2021 were included in this study to permit sufficient follow-up. The study received ethics approval from University Health Network, Research Ethics Board (REB #17-6284.7). Patient demographics and disease characteristics were obtained from the electronic health records. Baseline variables, such as age, gender, eastern cooperative oncology group performance status (ECOG PS), International Metastatic Renal cell carcinoma database consortium (IMDC) score, date of nephrectomy, baseline lactate dehydrogenase (LDH) level, and sites of distant metastasis, were collected. Pathological reports were carefully reviewed to determine the histological subtypes and presence of sarcomatoid or rhabdoid features. We also collected data on first- and second-line treatment settings, response to treatment (which was defined according to RECIST 1.1) (17), date of progression, and date of death were collected for survival analysis.

### Statistical analysis

Baseline demographics, and clinical and laboratory characteristics were described using absolute numbers and proportions for categorical variables and medians with interquartile ranges reported for continuous variables. For survival calculations, time from the start of treatment to the event of interest was used for PFS (disease progression) and OS (death from any cause). Survival analysis was performed using the Kaplan–Meier method (log-rank). Chi-square and Fisher’s exact test were used to evaluate response rates. Clinicopathological variables were analyzed for OS associations using a cox proportional-hazards model for univariable (UVA) and selected multivariable analysis (MVA). Hazard ratios (HR) with 95% CI were reported. All tests used P ≤ 0.05 for significance. IBM SPSS Statistics v28 (IBM; Armonk, NY, USA) was used to conduct statistical analyses.

## Results

### Patients’ characteristics

We identified 530 patients diagnosed with metastatic RCC at Princess Margaret Cancer Centre, Toronto between 2002 and 2021. A total of 69 (13%) were diagnosed with metastatic nccRCC either after nephrectomy (n = 31 [44.9%]) or tru-cut biopsy (n = 38 [55.1%]). The patients were treated with IO and/or TT. Median age was 54 years (range: 26–75 years), and 48 (69.5%) were males. Among the identified cohort, 42 patients (60.8%) had papillary, 10 (14.5%) had chromophobe, 14 (20.2%) had unclassified RCC, and 3 (4.3%) had an XP translocation. Sarcomatoid and rhabdoid features were found in 9 (13%) and 7 (10.1%) patients, respectively. Overall, as per the IMDC score, 15 (21.7%), 41 (59.4%), and 13 (18.8%) patients were categorized as good, intermediate, and poor risk patients, respectively. The most common metastatic sites were the lymph nodes followed by the lungs (Table 1).

**Table 1:** Patients and disease characteristics.

Variables	n = 69	%
**Age**		
Median (range)	54 y (26–75)	
**Gender**		
Male	48	69.5
Female	21	30.5
**Baseline KPS, median (range)**	90 (60–100)	
**Median LDH U/L, median (range)**	268 (137–566)	
**Histopathology**		
Papillary	42	60.9
Chromophobe	10	14.5
Unclassified	14	20.3
XP translocation	3	4.3
Sarcomatoid	9	13
Rhabdoid	7	10.1
**Distant metastasis sites**		
Lymph nodes	42	60.9
Lungs	39	56.5
Bone	23	33.3
Liver	13	18.8
Adrenal	7	10.1
Pancreas	4	5.8
Peritoneum	4	5.8
Brain	4	5.8
**IMDC score**		
Good risk	15	21.7
Intermediate risk	41	59.5
Poor risk	13	18.8
**Nephrectomy history**		
Nephrectomy at diagnosis	31	44.9
Cytoreductive nephrectomy	24	34.8
No nephrectomy	14	20.3

KPS: Karnofsky Performance Scale; LDH: lactate dehydrogenase; IMDC: International mRCC Database Consortium.

All patients (n = 69) received first-line treatment, with either IO-containing regimens (17.4%) or TT only (82.6%). Most commonly used regimens in the IO treatment group and the TT group were a combination of nivolumab + ipilimumab and sunitinib, respectively. We identified 36 (52%) patients who received the subsequent treatment. Of these, 8 (11.6%) patients received an IO-containing regimen, with nivolumab being the most often used treatment, and 28 (40.6%) patients received a TT, mostly everolimus (Table 2).

**Table 2:** Summary of key treatment regimens.

	n	%
**First-line therapy (n = 69)**		
Immunotherapy	12	17.4
Targeted therapy (TT)	57	82.6
**First-line treatment**		
Sunitinib	41	59.4
Ipilimumab and nivolumab	8	11.6
Sorafenib	5	7.2
Savolitinib	3	4.3
Pembrolizumib	3	4.3
Temsirolimus	2	2.9
Pazopanib	2	2.9
Pembrolizumib and axitinib	1	1.4
Other targeted therapy	4	5.8
**Second-line therapy (n = 36)**		
Immunotherapy	8	11.6
Targeted therapy	28	40.6
**Second-line treatment**		
Everolimus	13	18.8
Sunitinib	7	10.1
Nivolumab	7	10.1
Axitinib	2	2.9
Cabozantinib	2	2.9
Chemotherapy	2	2.9
Pembrolizumib and axitinib	1	1.4
Other targeted therapy	2	2.9

### Response and survival outcomes

**Figure 1:**
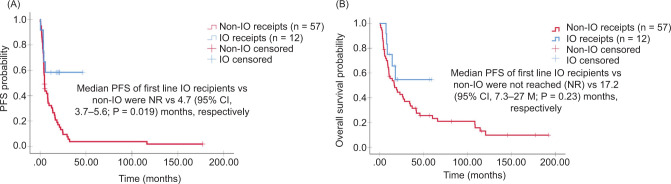
Kaplan–Meier survival curves of progression-free survival (PFS) and overall survival (OS) of the entire cohort in the first-line treatment. IO: immunotherapy.

**Figure 2:**
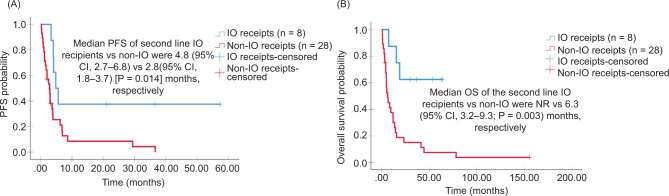
Kaplan–Meier survival curves of progression free survival (PFS) and overall survival (OS) of the entire cohort in the second-line treatment. IO: immunotherapy.

With a median follow-up of 116 months , the median PFS of the first-line IO recipients and non-IO recipients was not reached(NR) and 4.7 months (95% CI: 3.7–5.6; P = 0.019), respectively (Figure 1A). Median OS of the first-line IO recipients (n = 12) and non-IO recipients (n = 57) was NR and 17.2 months (95% CI: 7.3–27.0; P = 0.23), respectively (Figure 1B). ORR was significantly higher for patients treated with an IO regimen, 50% versus 12.5% in non-IO (P = 0.007) (Table 3). The ORR was low in patients with an unclassified RCC (8.3%) and XP translocation tumors (8.3%) and high in patients with papillary RCC (33.3%) (Table S1, Supplementary file). The median PFS of second-line IO recipients and non-IO recipients was 4.8 months (95% CI: 2.7–6.8) and 2.8 months (95% CI: 1.8–3.7; P = 0.014), respectively (Figure 2A). The median OS of the second-line IO recipients (n = 8) and non-IO recipients (n = 28) was NR and 6.3 months (95% CI: 3.2–9.3; P = 0.003), respectively (Figure 2B). ORR of IO recipients and non-IO recipients was 37.5% and 3.5%, respectively; P = 0.028 (Table 3). The ORR of IO recipients with papillary, unclassified, and chromophobe were 12.5% (Table S1). No interaction was observed between age, gender, IMDC, RCC subtypes, and survival outcomes.

**Table 3:** Response rate in the first- and second-line treatment settings.

Treatment line	Response type	IO	TT
First line	CR	2 (16.6%)	2 (3.5%)
	PR	4 (33.3%)	5 (8.7%)
	ORR	6 (50%)	7 (12.3%)
	SD	2 (16.6%)	28 (49.1%)
	PD	4 (33.3%)	22 (38.6%)
Second line	CR	1 (12.5%)	0
	PR	2 (25%)	1 (3.5%)
	ORR	3 (37.5%)	1 (3.5%)
	SD	2 (25%)	10 (35.7%)
	PD	3 (37.5%)	17 (60.7%)

IO: immunotherapy; TT: targeted therapy; CR: complete response; PR: partial response; ORR: objective response rate; SD: stable disease; PD: progressive disease.

## Discussion

Advanced nccRCC tumors are uncommon with diverse histological subtypes and variable prognosis (18). The study population included in our analysis was a representative of the patients with advanced nccRCC encountered in the real-world oncology practice. The majority of patients had either papillary, chromophobe, or unclassified nccRCC, consistent with the nccRCC literature’s reported epidemiology (3). Given the rarity of these tumors and the lack of prospective phase III trials to guide management, treatment decisions are extrapolated from the trials of ccRCC leading to increasing use of IO- and IO–TT-based combination approaches (11–15, 19). In the present single-center retrospective analysis, we found that IO-containing regimens were the effective treatment options for nccRCC in both first- and second-line treatment settings, compared to TT. The efficacy was higher in terms of better response rates and PFS (statistically significant), OS (statistically significant in second-line treatment setting).

The most common IO regimen used in the first-line treatment setting in the present study was ipilimumab + nivolumab. This IO combination was initially approved to treat metastatic ccRCC with poor or intermediate IMDC risk group based on the CheckMate-214 data. Unfortunately, this trial excluded nccRCC patients (11). The effectiveness of combining ipilimumab and nivolumab as a first-line treatment for metastatic nccRCC was reported by Gupta et al. in a study that included 18 patients (20). Among those patients, 6 (33%) had papillary RCC, 5 (28%) had chromophobe RCC, 3 (18%) had unclassified RCC, and 1 (5%) had XP translocation RCC. The ORR was 33.3%, including 3 (50%) for papillary RCC, 1 (33%) for unclassified RCC, and 1 (20%) for chromophobe RCC. The median PFS was 7.1 months (20). Tykodi et al. reported in the phase 3b/4 CheckMate 920 trial the activity of nivolumab + ipilimumab in 52 patients with advanced nccRCC (21). ORR was 19.6% (95% CI: 9.4–33.9). Two patients achieved complete remission (CR; 1 had papillary and another had unclassified pathology), 7 achieved partial response (PR) (4 had papillary and 3 had unclassified pathology), and 17 patients had stable disease (SD). The median PFS was 3.7 months (95% CI: 2.7–4.6), and the median OS was 21.2 months (95% CI: 16.6–not evaluable) (21).

Preliminary results of the phase II KEYNOTE-B61 study were presented at the European Society for Medical Oncology 2022. The study showed an ORR of 47.6% with an IO–TT regimen (pembrolizumab and Lenvatinib) (n = 82) in patients with advanced nccRCC. Median OS and PFS were not reached in this study (22). Lee et al. reported ORR for cohort 1, which included patients with papillary, unclassified, or translocation-associated RCC (23). The cohort had cabozantinib + nivolumab (n = 40) at 47.5% (95% CI: 31.5–63.9), with a median PFS of 12.5 months (95% CI: 6.3–16.4) and median OS of 28 months (95% CI: 16.3–not evaluable) (23).

McDermott et al. assessed the efficacy and safety of first-line pembrolizumab for the treatment of advanced nccRCC (24). The trial included 165 patients from the phase II KEYNOTE-427 study (cohort B). The authors reported an ORR of 26.7% for all patients. ORR by histology was 28.8% for papillary, 9.5% for chromophobe, and 30.8% for unclassified RCC. ORR was 35.3% and 12.1% for PDL1 CPS ≥ 1 and CPS < 1 status, respectively. Median PFS was 4.2 months (95% CI: 2.9 to 5.6); and median OS was 28.9 months (95% CI: 24.3 months–not evaluable) (24).

Metastatic RCC with Xp11.2 translocation/transcription factor E3 (*TFE3*) gene fusion is a rare distinct RCC subtype with aggressive behavior. It is now included in the classification of Microphthalmia-associated Transcriptional factor (MiT) family translocation RCC based on the World Health Organization (WHO) 2016 classification (3). Approximately 10% of patients had high PD-L1 expression (≥ 5% tumor cell membrane staining) (25). A multi-institutional study reported the efficacy of IO therapy in 43 patients with nccRCC, including three patients with metastatic RCC with XP translocation. The response was notable for one patient with PR, one patient with SD, and one patient with progressive disease (PD) (26). In our analysis, we identified one patient with metastatic XP translocation RCC, who received pembrolizumab in the first-line treatment setting and achieved complete remission that lasted for 46 months. During the course of IO therapy, he underwent cytoreductive nephrectomy that showed no viable tumor. AREN1721 is an ongoing phase II randomized clinical trial (NCT03595124) conducted in the United States, enrolling patients with metastatic RCC and an XP translocation to either a combination of nivolumab and axitinib or nivolumab alone to explore the role of IO therapy in this rare disease.

In the present study, we observed better efficacy of IO, compared to TT in the second-line treatment setting (n = 36) in patients who had progressed on previous TT (ORR 37.5% versus 3.5%) with a statistically significant P-value. Significant improvements were also noted in median PFS and OS with IO-based treatments. The most common IO therapy used in our study was nivolumab.

Nivolumab was approved to treat patients with metastatic ccRCC who failed antiangiogenic therapy based on the CheckMate-025 trial. This trial excluded patients with nccRCC (27). The efficacy of nivolumab in patients with advanced nccRCC who had undergone at least one prior treatment in the metastatic setting was demonstrated in a prospective trial conducted by Vogelzang et al. (28). This trial included a cohort of 44 patients from the phase IIIb/IV CheckMate 374 trial. The cohort had a median follow-up duration of 11 months, ranging from 0.4 to 27 months. The ORR was found to be 13.6% (95% CI: 5.2–27.4). The median PFS was determined as 2.2 months (95% CI:1.8–5.4). Additionally, the median OS was observed as 16.3 months (95% CI: 9.2–not evaluable) (28).

Koshkin et al. reported the first retrospective analysis for the survival outcomes of 41 patients with nccRCC treated with nivolumab after the failure of TT (29). Among those patients, 16 (39%) had papillary RCC, 5 (12%) had chromophobe RCC, and 14 (34%) had unclassified RCC. With a median follow-up time of 8.5 months, 7 (20%) had PR and 10 (29%) had SD. Responses were observed in 14% papillary RCC and 36% in unclassified RCC. In the entire cohort, the median PFS was 3.5 months, and the median OS was NR (29). These results suggested that patients who do not receive IO in the first-line treatment setting should be offered IO in the second-line treatment setting.

We acknowledge several limitations in this study. First, it was a nonrandomized, retrospective analysis evaluating a small number of patients with heterogeneous characteristics because of the rarity of the disease. Therefore, the survival benefit shown in this study could be related to other unidentified variables. In addition, this study could not analyze the effectiveness of IO combinations versus IO with TT because of the small number of patients enrolled. Second, the benefit of IO therapy appeared to be driven predominantly by the papillary subgroup, and the effectiveness in other smaller subgroups remained inconclusive. Third, in the last decade, significant advancements have been observed in the treatment of advanced RCC. This is primarily due to the introduction of novel targeted treatments, innovative immunotherapies, and improved supportive care. Half of our cohort received treatment prior to 2015. Consequently, this could have an impact on their survival outcomes. Finally, sunitinib was the most commonly administered TT in the present study group. Cabozantinib, a treatment that showed recently significant prolongation of PFS in papillary RCC patients, compared to sunitinib, was not used in the first-line treatment setting. The absence of cabozantinib could impact the survival outcomes of the TT arm.

## Conclusions

While the number of patients included in the present retrospective review was small, our analysis suggested that advanced nccRCC with variable histological subtypes showed potential responsiveness to IO-containing regimens. The outcomes of our study could assist clinicians in selecting the treatment approach for these rare tumors while waiting for more conclusive prospective randomized data.

## Figures and Tables

**Table S1:** Best response to first- and second-line treatment settings based on histopathological subtype.

Treatment line	Response type	IO therapy					Targeted therapy				
		Papillary	Chromophobe*	Unclassified	XP translocation	Total response	Papillary	Chromophobe	Unclassified	XP translocation	Total response
First	CR	1	0	0	1	2 (16.6%)	0	2	0	0	2 (3.5%)
	PR	3	0	1	0	4 (33.3%)	4	0	1	0	5 (8.7%)
	ORR	4 (33.3%)	0	1 (8.3%)	1 (8.3%)	6 (50%)	4 (7%)	2 (3.5%)	1 (1.8%)	0	7 (12.3%)
	SD	1	0	1	0	2 (16.6%)	19	5	4	0	28 (49.1%)
	PD	3	0	0	1	4 (33.3%)	11	3	7	1	22 (38.6%)
Second	CR	0	0	1	0	1 (12.5%)	0	0	0	0	0
	PR	1	1	0	0	2 (25%)	1	0	0	0	1 (3.5%)
	ORR	1 (12.5%)	1 (12.5%)	1 (12.5%)	0	3 (37.5%)	1 (3.5%)	0	0	0	1 (3.5%)
	SD	0	2	0	0	2 (25%)	8	1	1	0	10 (35.7%)
	PD	0	3	0	0	3 (37.5%)	11	2	3	1	17 (60.7%)

IO: immunotherapy; CR: complete response; PR: partial response; ORR: overall response rate; SD: stable disease; PD: progressive disease. *No chromophobe patients were identified in the IO group in the first-line treatment setting.
